# A Study of the Association between Primary Oral Pathologies (Dental Caries and Periodontal Diseases) Using Synchrotron Molecular FTIR Spectroscopy in View of the Patient’s Personalized Clinical Picture (Demographics and Anamnesis)

**DOI:** 10.3390/ijms25126395

**Published:** 2024-06-10

**Authors:** Pavel Seredin, Tatiana Litvinova, Yuri Ippolitov, Dmitry Goloshchapov, Yaroslav Peshkov, Vladimir Kashkarov, Ivan Ippolitov, Boknam Chae

**Affiliations:** 1 Department of Solid-State Physics and Nanostructures, Voronezh State University, 394018 Voronezh, Russia; centr_rus_yaz@mail.ru (T.L.);; 2 Psycholinguistic Textual Modelling Lab, Voronezh State Pedagogical University, 394043 Voronezh, Russia; 3 Department of Pediatric Dentistry with Orthodontia, Voronezh State Medical University, 394006 Voronezh, Russia; 4 Pohang Accelerator Laboratory, Beamline Research Division, Pohang 37673, Republic of Korea

**Keywords:** synchrotron FTIR, gingival crevicular fluid, personalized clinical picture

## Abstract

In this exploratory study, we searched for associations between the two most common diseases of the oral cavity—dental caries and periodontal diseases—taking into account additional factors, such as personalized clinical pictures (the individual risk factors of the patient), based on the method of a multivariate data analysis of the molecular changes in the composition of human gingival crevicular fluid (GCF). For this purpose, a set of synchrotron Fourier-transform infrared spectroscopy (FTIR) spectra of gingival crevicular fluid samples from patients with different demographics, levels of dental caries development and periodontal diseases, and the presence/absence of concomitant chronic diseases were obtained and analyzed. Using a set of techniques (v-, F-, Chi-square tests; a principal component analysis (PCA); and the hierarchical clustering of principal components (HCPCs)) implemented in the R package FactoMineR allowed us to assess the relationship between the principal components (PCs) and characteristics of the respondents. By identifying the features (vibrational modes in the FTIR spectra) that contribute most to the differentiation of the spectral dataset, and by taking into account the interrelationships between the patients’ characteristics, we were able to match specific biological markers (specific molecular groups) to the two factors of interest—two types of oral pathologies. The results obtained show that the observed changes in the quantitative and qualitative composition of the modes in the infrared (IR) spectra of the GCF samples from patients with different dental caries developments and periodontal diseases present confirm the difficulty of identifying patient-specific spectral information. At the same time, different periodontal pathologies are more closely associated with other characteristics of the patients than the level of their caries development. The multivariate analysis performed on the spectral dataset indicates the need to take into account not only the co-occurrence of oral diseases, but also some other factors. The lack of this consideration (typical in lots of studies in this area) may lead to misinterpretations and consequently to a loss of data when searching for biological markers of certain oral diseases.

## 1. Introduction

The prevention of oral diseases is of great importance due to the large influence of dental status on all spheres of human social activity at every age [1,2]. It has been proven that dental caries and periodontal diseases are the most widespread non-infectious human diseases, leading to negative consequences for people’s quality of life, including teeth loss [3,4]. Therefore, the early diagnosis of the initial forms of these diseases is one of the top-priority research areas [5,6,7].

An analysis of the results of works where the processes occurring in the oral cavity are considered at the molecular level allows us to reasonably assert that the healthy state of dental tissue is determined by a strictly defined balance in the organ–mineral complex of the tooth and pulp, biofilm formed on the tooth surface, oral fluids, and periodontal tissues [8,9,10]. At the same time, the state of the dental biofilm is the main biological determinant affecting the development of dental caries and periodontal diseases [11,12,13,14]. Therefore, the development of methods for the early screening of both types of diseases can be based on the molecular identification of specific compounds in the oral fluids conjugated with the organo–mineral complex of the teeth and periodontal tissues [15,16,17,18,19]. In this regard, gingival crevicular fluid (GCF) is the most attractive bioanalyte, whose composition, in the presence of inflammation in the body, may contain specific cells, components of serum, connective tissue, epithelium, and microbial flora [18,20,21,22]. Therefore, the molecular content of GCF represents a promising source of biomarkers [21,22,23,24,25] that may prove to be a source of important information, for example, for the assessment of the expression of genes encoding superoxide dismutase 1 (SOD1), glutathione peroxidase 1 (GPX1), and thioredoxin 1 (TXN1) in the saliva and gingival tissue of patients with periodontitis [26] or to determine the concentrations of selected non-enzymatic antioxidants in the gingival fluid and saliva of patients diagnosed with periodontitis [26].

It should be noted that molecular changes in the composition of bioanalytes during the development of oral pathologies also depend on individual human parameters (age, sex) [4,27,28,29] and patients’ personalized clinical pictures (state of dentition, periodontal tissues, and anamnesis) [17,18,30,31]. However, the currently used analytical tests for screening oral pathologies (dental caries and periodontal diseases) [21,32,33,34,35] are based on data sampling with averaging over a group of patients, which does not allow for a personalized view of the disease course to be obtained. Moreover, long-term studies have shown that, although the two major oral diseases sometimes seem unrelated, dental caries shows different, although sometimes controversial, associations with periodontal disease [3,4]. Recent studies have demonstrated a small positive relationship between the clinical and microbiological indicators of both oral diseases [7,12,13,14]. But to understand the nature of this relationship and these disease interactions, the development of new strategies is needed to reveal the changes in the GCF’s composition in terms of the development level of oral pathologies, taking into account the patient’s personalized clinical picture [16,27,36].

Such a classification can be obtained using molecularly sensitive diagnostic methods, reflecting years of actual experience in disease research, and the detection of specific biomarkers characteristic of caries and periodontal disease development. In this context, Fourier-transform infrared (FTIR) spectroscopy, which allows for the extraction of biochemical information on the composition of the analyte, is the optimal method to study GCF characteristics [19,24,36,37]. FTIR spectroscopy is the golden standard in studies of the molecular composition of samples of a biological nature. As was previously shown, using IR spectroscopy it is possible to determine glucose levels, prediabetes [31,38], the level of periodontitis [39], oncologic markers [40], and predispositions to caries, as well as perform the monitoring of its development [16]. An advantage of FTIR spectroscopy is that, due to the fact that this method is non-invasive, it does not affect the object under study, thus there appears to be a possibility of studying the changes in the molecular composition of oral cavity fluids during the development of a pathology, including those that depend not only on the primary structure of the protein molecules composing the fluid but on their secondary structure as well, i.e., on their spatial structure [41].

The combination of synchrotron radiation and FTIR spectroscopy methods allows one to achieve the best spectral and lateral resolution exceeding (by an order of magnitude) the level of laboratory FTIR instruments, which is extremely important for the analysis of samples collected in macro quantities [42,43,44,45,46]. 

Given the multifactorial nature and complexity of both diseases, in order to identify associations between them, it is necessary to search for the structure in the collected data, using the methods of multivariate analysis, to identify the factors that influence the variation in the spectra, including the periodontal state and a stage of caries development, anamnesis and demographic characteristics [4,7,10,47,48]. 

It should be noted that when predictive models for diagnosing the development of oral diseases based on the analysis of the spectral information (the molecular spectra of various analytes) do not usually take into account the effect of associated factors (information about the patients) [49,50,51,52]. This, in turn, may affect the interpretation of the obtained results. Considering that oral diseases are usually associated with other pathologies, demographics, etc., a simultaneous search for relationships of spectral features with patients’ personalized factors is necessary [19,53,54,55]. 

Therefore, the aim of our paper is the search for associations between the two most common types of oral diseases (dental caries and periodontal diseases) taking into account patients’ personalized information, based on identifying the changes in the molecular composition of GCF using synchrotron molecular FTIR spectroscopy and multidimensional data analysis techniques.

The novelty of our research is that we applied the multivariate data analysis techniques with the use of a set of functions directed at the ascertainment of the relationships for new combined variables (components) with these or those factors related to patients’ personalized clinical pictures. These methods have not yet been widely applied for an exploratory analysis of spectral datasets, however, they are well documented [56] and have been used for the ascertainment of the latent structure in datasets in different areas of knowledge. An advantage of the applied methodology is in the fact that it is implemented in the form of the publicly available libraries and replicable. A specific feature of our work is the use of exclusively open instruments for the data analysis written in R, a free software environment for statistical computing and graphics.

## 2. Results and Discussion

### 2.1. FTIR-Spectroscopy

**Figure 1:**
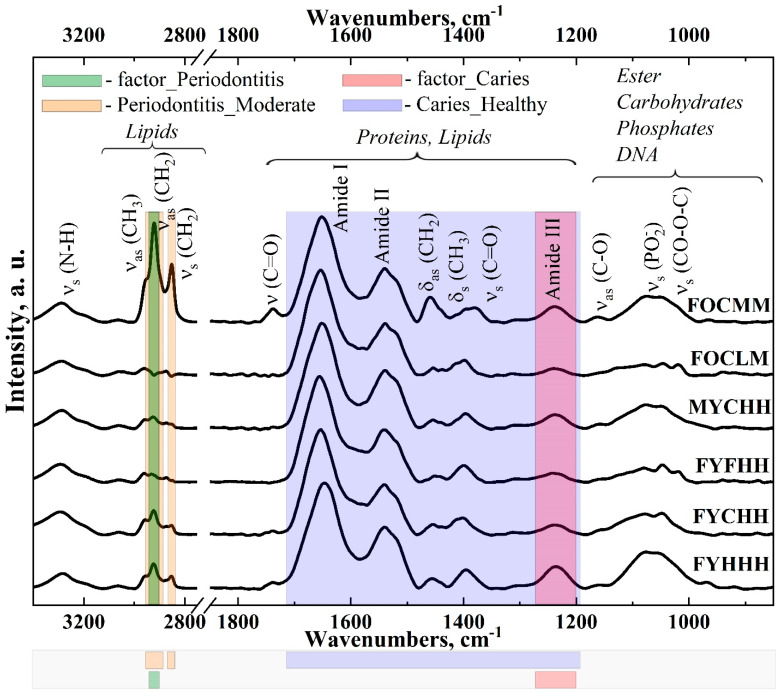
FTIR spectra of the gingival crevicular fluid samples from the patients with varying stages of caries development, periodontal status and personal history in the ranges of 3400–2800 cm^−1^ and 1800–870 cm^−1^. Information about the patients is coded in the sample names in the following order: gender, age, caries development stage, periodontal status and anamnesis.

Figure 1 shows a selection of infrared spectra of GCF samples in the wave number ranges of 3400–2800 cm^−1^ and 1800–870 cm^−1^. These regions of the spectrum were chosen as the main absorption bands are located there, for which the change of intensities depending on the caries development stage, periodontal status and personal history of a patient was recorded.

Figure 1 shows the averaged spectra characteristic of the samples from the patients with a specific periodontal status, caries development stage and personal history. Each of the individual spectra in the selection (see FTIR technique) contained the same set of absorption bands corresponding to the characteristic molecular bonds. A slight dispersion of the intensity of the main vibrational modes was observed for the spectra in the samples, which is clearly related both to the patients’ individual characteristics and the conditions of the FTIR experiment. The preliminary use of multivariate data analysis methods allowed us to take this dispersion into account in further analysis. The investigation of molecular composition features was performed using the averaged spectra.

The results obtained show (see Figure 1) that GCF has a unique FTIR spectrum pattern, and three regions can be conventionally distinguished in the spectra in which the main absorption bands are located: 3400–2800 cm^−1^, 1800–1200 cm^−1^ and 1200–870 cm^−1^.

The group of vibrational bands localized in the FTIR spectra between 3400 and 3150 cm^−1^ refers to the presence of Symmetric N-H str molecular groups of proteins in GCF samples [15,37,57,58,59,60]. The maxima in the range of 3100–2800 cm^−1^ refer to the presence of lipid and fatty acid molecular groups in the CH_2_, CH_3_ samples [29,58,61]. It should be noted that the relative intensity of these vibrational bands in the spectra obtained from the healthy group is small [37], but it changes significantly in the presence of pathology. In addition, as it was shown earlier, asymmetric and symmetric valence vibrations associated with lipids -CH_2_ and -CH_3_, and located in the region of 2800–3100 cm^−1^ of the GCF IR spectra allow discriminating the patients with different periodontal diseases [55]. It is well known that lipid oxidation is markedly increased during periodontal inflammation [55], which is reflected in the intensity of the Olefinic band = CH of around 3000 cm^−1^.

At the same time, it is necessary to note the presence of an absorption band in some FTIR spectra of GCF in the region of 1765–1725 cm^−1^ (Figure 1). In accordance with the data reported in [16,37,48,57,59,62], this mode represents the >C=O vibrations and can be associated with the ester carbonyl group. Note that the presence of esters in a human dental hard tissue during dental caries was shown earlier in a number of works [39,63,64]. Their authors pointed to the fact that esters are more frequently found in a carious tissue than in an intact one [65].

The group of the vibrational modes in the region of 1710–1190 cm^−1^ belongs to proteins, amino acids and CH_2_/CH_3_ bonds of various molecules. The following bands attributable to proteins can be distinguished in this group: Amide I (C=O stretch vibr. in the region 1725–1590 cm^−1^), Amid II (N-H bend and C-N stretch in the region 1590–1500 cm^−1^) and Amid III (C-N stretch, N-H bend in the region 1350–1190 cm^−1^), which under certain conditions are potentially significant for the diagnosis of various pathologies [41,66,67,68,69]. A detailed examination of the spectra in Figure 1 shows that the profile of Amide I, Amide II, and Amide III bands is transformed, which is associated with the changes in the secondary structure of the proteins that make up GCF [15,24,30,36,48,58]. The changes in the spatial and conformational structure, in the presence of concomitant factors, may be associated with different pathological processes in the human body.

The variations of CH_2_/CH_3_ groups located in the region of 1480–1350 cm^−1^ [70] are associated with the Amino acid side chains, lipids & Proteins Fibrinogen/methyl bending of the amino acid side chains, lipids and proteins [30,71,72]. GCF contains a significant amount of serum and cellular structures, nucleic acids, including Deoxyribonucleic acid (DNA) and Ribonucleic acid (RNA). Tajmir et al. in their work [73] showed that human serum albumin, interacting with nucleic acids under certain conditions, changes and the characteristic factor of this process is the rearrangement of the vibrational band in the IR spectrum in the region of 1220–1210 cm^−1^. Modern studies of inflammatory processes of a bone tissue show that this spectral region also displays the oscillations attributed to antibodies to nuclear antigens (ANA) [52].

The vibrational band in the region of 1160–1152 cm^−1^ can be attributed to carbohydrates [30,59,72]. An increase in the level of carbohydrates in the oral fluid, as it was shown in our previous work [16], can be considered as a sign of the carious process development.

The group of the vibrational bands in the spectra, located in the range of 1130–850 cm^−1^, belongs to molecular bonds related to the phosphates, glycerophosphates and phospholipids [60,74], as well as carbohydrates and the DNA derived structures. This group of the vibrational bands shows significant rearrangements depending on the type of pathology (see Figure 1). Consistent with the findings reported in [55], the bands at 950 cm^−1^ and 1190 cm^−1^ are significant markers for the differentiation of various periodontal diseases.

The results of analyses of similar vibrations and intensity ratios between them in the IR spectrum of GCF are in good agreement with the findings reported in [75].

The analysis of the spectral selection presented in Figure 1, taking into account the information about the participants, allows one to state that the intensity and spectral position of vibrational modes active in the GCF spectra whose association with molecular groups is given above change. This occurs due to the shifts in the GCF composition and transformation of lipids, carbohydrates proteins and the DNA structures content in the presence of periodontal inflammatory processes, caries development stage, chronic diseases stage. Moreover, the presence of inflammation and caries is also related to the changes in the concentration of cytokines, enzymes and metabolites in GCF. These findings are consistent with the results of the previous studies with the use of FTIR [15].

**Figure 2:**
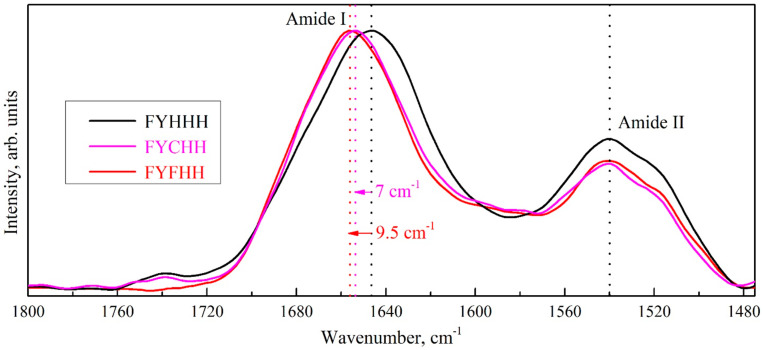
Amide profiles in the FTIR spectra of gingival crevicular fluid of the patients with different stages of caries development. Information about the patients is coded in the sample names in the following order: gender, age, caries development stage, periodontal status and anamnesis.

**Figure 3:**
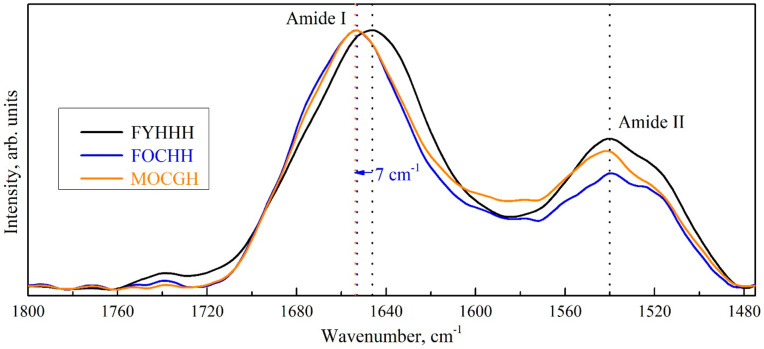
Amide profiles in the FTIR spectra of gingival crevicular fluid of the patients with a different periodontal status. Information about the patients is coded in the sample names in the following order: gender, age, caries development stage, periodontal status and anamnesis.

All these changes in the GCF composition are reflected in the spectral response and result in the conformational changes (transformation) of the secondary structure of proteins, lipids, and DNA. At the same time, significant shifts of IR vibrations bands related to lipids, proteins and acids may occur. Thus, it is clearly seen that at different stages of caries development (Figure 2), as well as under different periodontal conditions (Figure 3), the main amide bands Amid I and Amid II, which provide the information both about the protein content and their secondary structure, demonstrate not only a transformation of the profile, but also of the spectral position. A similar fact has been previously observed in our studies devoted to the shifts in the composition of the dental biofilm [61,76].

### 2.2. Multivariate Analysis

In order to understand and classify the changes occurring in the FTIR spectra of GCF samples and to identify oral disease associations taking into account the personalized clinical picture of the patients, the collected spectral dataset was processed using a set of multivariate analysis techniques.

The proposed set of multivariate analysis methods allows one to take into account, firstly, the interrelation of various characteristics of the participants, and secondly, to establish the links between the patients’ biological markers and states which can be further used to build more accurate and biologically justified predictive models for the diagnostics of oral diseases in future research. In the existing body of research aimed at classifying the spectra and constructing predictive models using multivariate analysis methods [77,78], two groups of participants—healthy and ill ones—are typically considered, and the target function of the classifier is to determine the class of the sample (healthy/diseased), while the relationship between comorbid pathologies and the participants’ individual characteristics are usually not taken into account. Although, for example, authors of [78] observed differences in salivary metabolites among the dentition stages, which may be related both to physiological and social behavioural changes during the prepubertal period even in the healthy respondents.

The use of multivariate analysis techniques to process the FTIR spectra is necessary to identify subtle differences in the spectral dataset that are not obvious when only the absorption profile is analyzed due to the large number of overlapping oscillations. At the initial stage, the entire spectral range in which the vibrational modes identified in the molecular analysis step are located was initially used to extract some valuable information and to facilitate the interpretation of the dataset.

**Figure 4:**
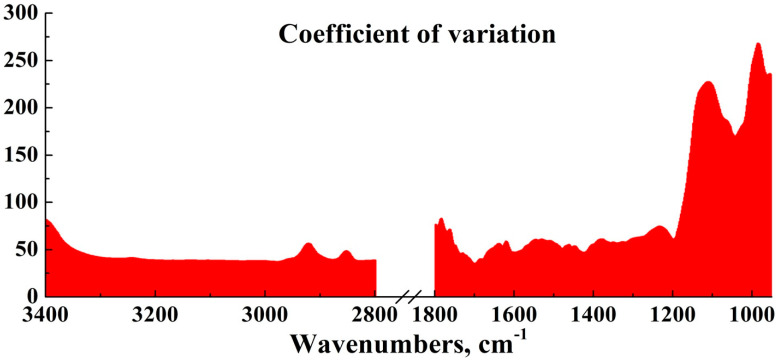
Coefficient of variation of the features (intensities in the wave numbers).

At the first step of the analysis, we estimated the variance of the features (intensities in the wave numbers) by calculating their coefficient of variation (Figure 4).

The mean value of the coefficient of variation in the dataset is ~74.06%, with a standard deviation of ~58.54%. The median value of the coefficient of variation is ~52.69%, with the highest values of this coefficient (over 100%) observed in the ranges from 1172 to 950 cm^−1^. This is due to the fact that the group of oscillatory bands experiences significant transformations in their intensity depending on the type of pathology. 

To investigate the relationship between the categorical variables reflecting the stage of caries development, periodontal status and some other characteristics (anamnesis, age, gender), a number of statistical tests were performed using R package lsr. Those included the Chi-square test to assess the presence of an association between the categorical variables, and the Cramer test to assess the strength of such associations (if any). The analysis showed that the factor Caries (factor_Caries, i.e., caries development stage) was not associated with the other categorical variables in our dataset. At the same time, the factor Periodontal status (factor_Periodontitis) was associated with Health status (factor_Anamnes, *p* = 0.0182) and Age (factor_Age, *p* = 0.034).

**Figure 5:**
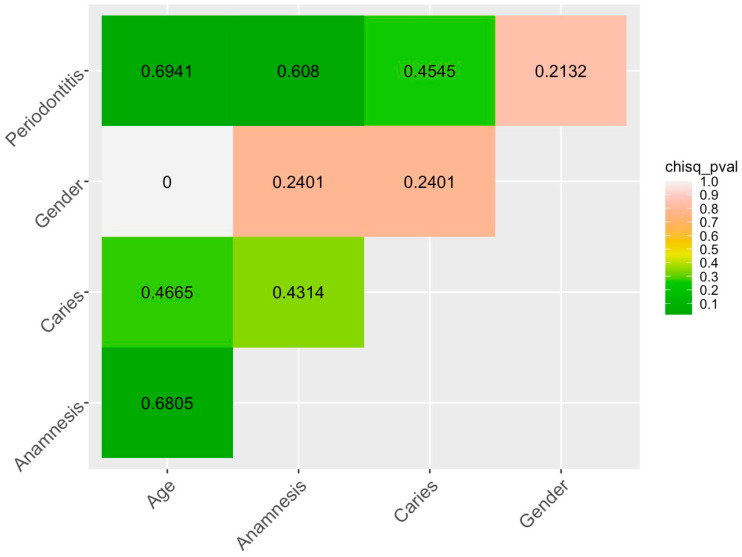
Correlations between the factors describing the patients’ characteristics (the Cramer’s test value is given in the center of the squares; a color bar represents the significance level of the Chi-square test).

A visual representation of the relationship between all the categorical variables describing the patients is shown in Figure 5, where the Cramer test value is indicated numerically and the Chi-square test *p*-value is colored.

**Figure 6:**
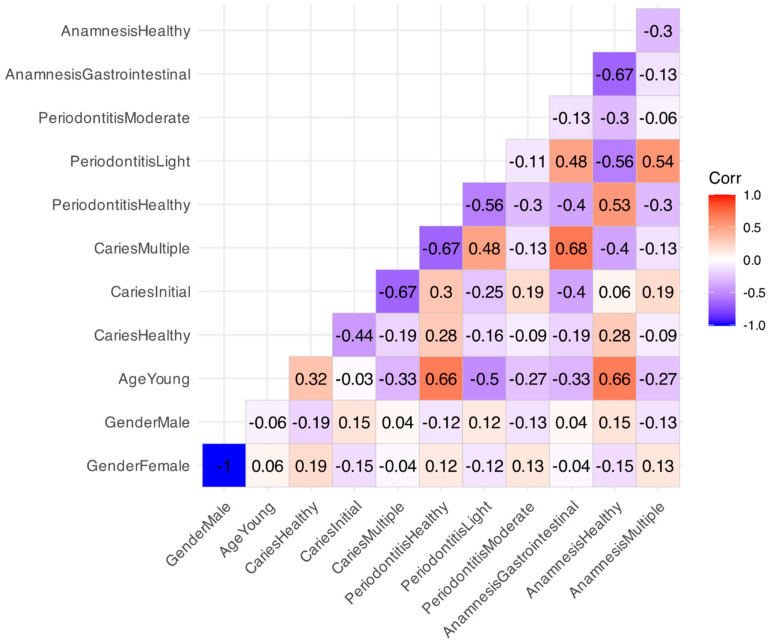
Correlation matrix of the relationship between all the levels of all the factors describing the patients characteristics: gender, age, caries development stage, periodontal status, anamnesis. The factor Age has two levels: Young (patients of 25–44 y.o.) and Old (patients of 45–60 y.o.). The factor Caries has following levels: Healthy, Initial, Multiple and Fissure. The factor Periodontitis has following levels: Healthy, Gingivitis, Light and Moderate. The factor Anamnesis has levels Healthy, Gastrointestinal, Cancer and Multiple.

In addition to the analysis of the association between the factors, we also analyzed the association between the factor levels using Pearson correlation coefficient. The values of the correlation coefficients between all the levels of all the factors are presented in Figure 6 (calculation of the correlation coefficients and visualization of the correlation matrix was carried out using the R package ggcorrplot). Note that we calculated the correlations between all the pairs of variables.

Note that both types of the analyses are complementary: a full correlation analysis of factor levels assumes that they are treated as independent variables (as most regression models do), unlike the Cramer’s V-test.

Thus, the analysis performed (Figure 5 and Figure 6) showed a complex picture of the interrelations of the patients’ characteristics in our dataset. Thus, in the absence of a relationship between factor_Caries and factor_Periodontitis, there is a correlation between the individual levels of these factors: a negative correlation between Multiple Caries and Periodontal Health, a positive correlation between Multiple Caries and Light Periodontitis. We witness the important role of factor_Age: there is a moderate positive correlation between the young age (Age_Young) and absence of caries (Caries_Healthy), and a strong positive correlation between the level of periodontal status Periodontal_Healthy and general health (level Anamnesis_Healthy).

There is also a strong positive correlation between multiple caries (Caries_Multiple) and gastrointestinal illnesses (Anamnesis_Gastrointestinal), and a moderate correlation between light periodontitis (Periodontitis_Light) and gastrointestinal illnesses (Anamnesis_Gastrointestinal). The patient’s health (Anamnesis_Healthy) is correlated positively with periodontal health (Periodontal_Healthy). There is also a positive correlation between the general health (Anamnesis_Healthy) and the absence of dental caries (Caries_Healthy). Light periodontitis (Periodontitis_Light) is positively correlated with multiple pathologies (Anamnesis_Multiple). 

In general, various periodontal pathologies are more strongly correlated with the other patients’ characteristics than caries development stage. 

The established correlations require a detailed analysis of the pattern of the relationships between certain characteristics of the spectra of the GCF samples and the patients’ characteristics. To assess the main sources of variation in the data, a multivariate analysis was performed, namely principal component analysis (PCA) with the subsequent establishment of the relationship between the identified principal components (PCs) and the characteristics of the spectra and the patients. The intensities in the spectra at the given wave numbers for each sample were considered as active variables, while characteristics of the patients (presented as factors and their levels) were used as supplementary categorical variables not involved in the analysis but used for the interpretation (supplementary qualitative variables).

**Figure 7:**
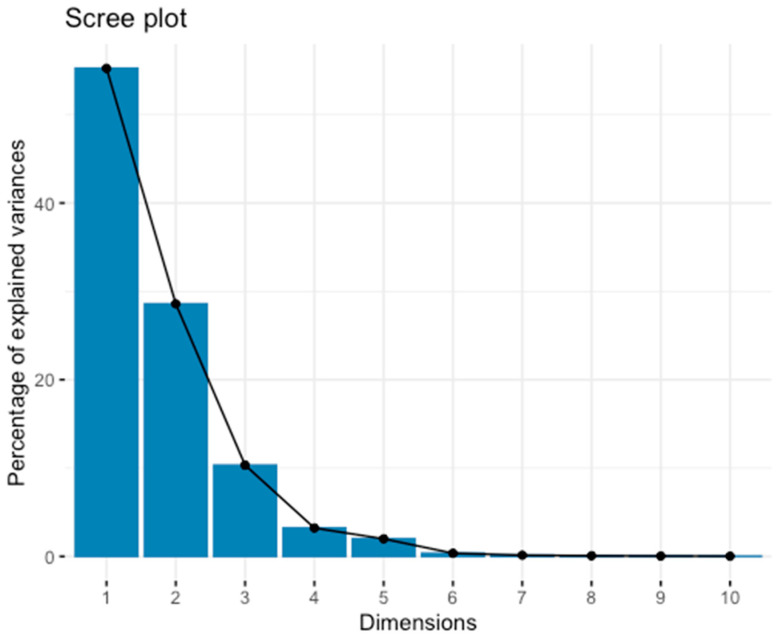
Percentage of the variation explained by first 10 principal components.

At the first stage of the analysis, the number of the principal components was determined. There are different approaches to PCs number determination. According to the Kaiser Criterion, the components with eigenvalues greater than 1 should be considered for further analysis. In our analysis, the first 7 components, which together explain 99.84% of the variation, meet these criterion (Figure 7).

A common empirical practice for choosing the number of the components is the elbow rule, that is, cutting off those followed by a decrease in the percentage of explained variation in the screeplot plot [79]. “Elbow” means that the remaining variation is distributed among a lot of components and is irrelevant to obtain a “low-dimensional” representation of the data. 

For further analysis we selected the first 5 components based on the two criteria described above.

For the categorical variables, an anova model with one factor is done for each dimension; the coordinates of the individuals are explained by the categorical variable. An F-test is conducted to see whether the variable has an influence on the dimension, and *t*-tests (with contrast sum alpha_i = 0) are conducted category by category. The v-test is used to detect contribution of a given variable in the category. 

The analysis was performed using R package FactoMineR.

The factor Caries is shown to be associated with the PC 1 (i.e., the most important) (R^2^ = 0.209, *p* < 0.05).

The v-test indicates an association of Caries_Healthy level with the PC 1 (v-test =1.94, *p* < 0.05). 

**Figure 8:**
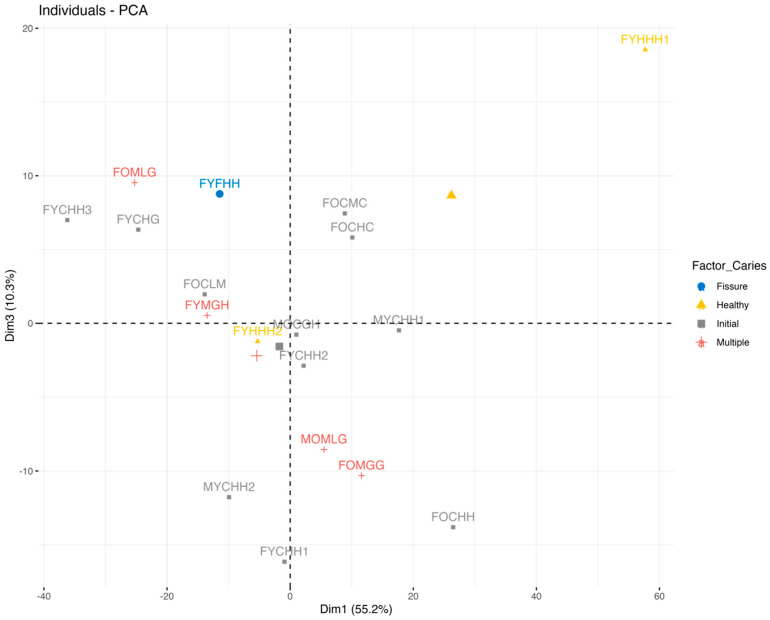
PCA (1st and 3rd components) with the visualization of the differences in the samples location by the factor Caries.

PCA (1st and 3rd components) with the visualization of the differences in the samples location by the factor Caries is presented in Figure 8.

As Figure 8 shows, for the first component, there is a separation between the caries-healthy participants (Caries_Healthy) and the individuals with different stages of caries development. Thus, the presence/absence of caries is the main source of variation in our dataset.

Factor_Anamnesis has the highest R^2^ value for PC 2 (0.41, *p* < 0.05). The v-test indicates a close association of the Anamnesis_Cancer (v-test = 2.64, *p* = 0.004) with the PC 2. This observation indicates the need to consider aggravating medical history in dental research.

Factor_Periodontitis has the highest R^2^ value for PC 4 (0.4064, *p* < 0.05), as well as factor_Age (0.2348, *p* < 0.05) and factor_Caries (0.205, *p* < 0.05), which confirms the complex interrelationship of these factors previously shown when analyzing the interrelationships of the patients’ characteristics.

For factor_Periodontitis, the v-test shows the strong association between PC 4 and Periodontal_Gingivitis level (v.test = −2.247, *p* = 0.0193). The v-test confirms the association of both levels of the factor_Age with PC 4 (v.test = −1.99 for Age_Old and v-test = −1.99 for Age_Young respectively, *p* = 0.041). For factor_Caries, no association of its individual levels with the PC 4 was recorded.

Factor_Periodontitis explains the highest percentage of variation (R^2^ = 0.778, *p* < 0.00001) for PC 5, as well as factor_Anamnesis (R^2^ = 0.499, *p* = 0.018) and factor_Age (R^2^ = 0.228, *p* < 0.05). The v-test confirms the association of both factor_Age levels with PC 5 (v-test = −1.99 for Age_Old and v-test = 1.99 for Age_Young respectively, *p* < 0.05), strong association of PC 5 with Periodontitis_Moderate (v-test = −3.53, *p* < 0.00001), Anamnesis_Healthy (v.test =2.0798, *p* < 0.05) and Anamnesis_Cancer (v.test = −2.53, *p* < 0.001).

**Figure 9:**
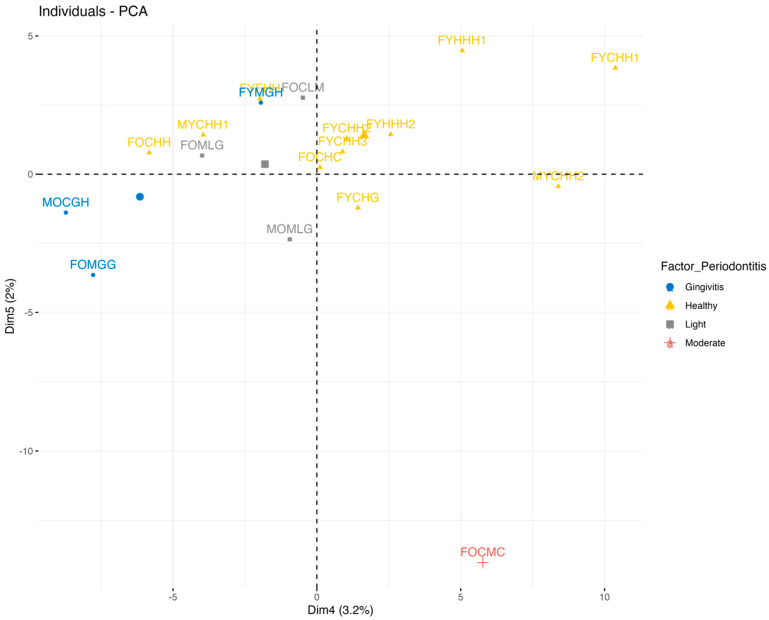
PCA (PC 4 and PC 5) with samples colored by periodontal status of patients (factor_Periodontitis).

The PCA (PC 4 and PC 5) with the visualization on factor_Periodontitis is shown in Figure 9.

Figure 9 clearly shows the clusters of the samples by the patients with various periodontal pathologies, which indicates that it is promising to build predictive models (classifiers) aimed at diagnosing different types of periodontal diseases using appropriate biomarkers. It is likely that the biomarkers of different periodontal pathologies are more pronounced at the spectral level than the different stages of caries development, where the healthy/diseased opposition is observed. 

The dimdesc function of FactoMineR packages allowed us to calculate the correlation coefficient between a variable and a dimension with follow-up significance test. 

Loadings (i.e., standard coordinates) are not calculated by FactoMineR (it returns the principal coordinates), so we calculated them by dividing the coordinates of the variables on a dimension by this dimension’s eigenvalue’s square root.

**Figure 10:**
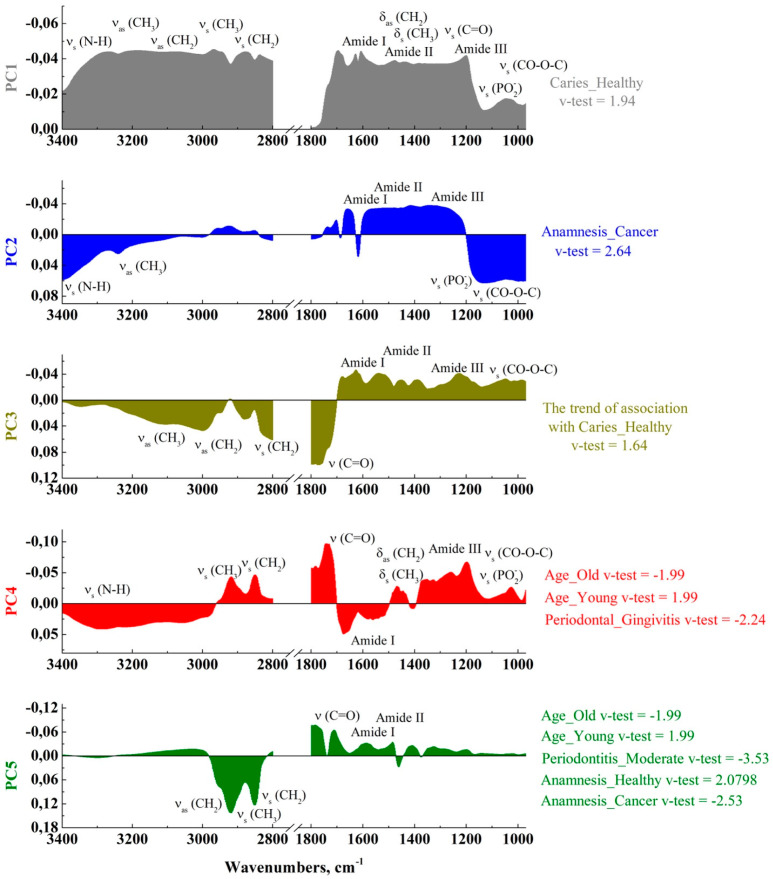
Loading plots for the principal components PC 1–5 and their relationships with factor levels.

Figure 10 shows the component loadings, i.e., the correlations of features (intensities in the wave numbers) with the selected components, and the corresponding PC associations with the corresponding factor levels.

In Figure 10, the loading plots are presented for each PC. They visualize the set of maxima that make the main contribution to the observed differences in the GCF samples with respect to the patient’s personalized picture. Also, in the PCA loading plots, for each PC its relationship with the factor levels is highlighted and, for the significant maxima, the corresponding functional molecular groups are indicated based on the literature data.

As we have shown previously, factor Caries is associated with the PC 1. At a significance level of *p* < 0.05, the intensities in the range of 1220–1200 cm^−1^ correlate with this component, which corresponds to the PO^2^- asymmetric stretching observed for the phosphodiesters and nucleic acids molecules [15,19,24,37,48]. Tajmir et al. in [73] showed that human serum albumin interacts with nucleic acids under certain conditions. In this case, a rearrangement of the vibrational band in the region of 1220–1210 cm^−1^ is observed in the IR spectrum. Modern studies of inflammatory processes of a bone tissue suggest that this spectral region also shows antibodies to nuclear antigens (ANA) [52].

Regarding the individual levels of factor Caries, all the detected relationships are related to the Anamnesis_Healthy. The most significant relationships between PC 1 associated with a given factor level and features were found for the intensities in the range of 1280–1200 (*p* < 0.01), which corresponds to the maxima of the Amid III band (C-N stretch, N-H bend in the region 1350–1190 cm^−1^).

The v-test statistics indicates the relationship between Periodontitis_Moderate level and PC 5, and the correlation test implemented in the dimdesc function shows the relationship between the intensities in the range of 2931–2844 cm^−1^ and this component, which corresponds to the symmetric and asymmetric stretching of the C-H groups CH_3_, CH_2_ attributed to the lipids and the fatty acids chains [15,24,37,48].

In addition to the above analysis, using FactoMineR, dist values were calculated, which provides some insight into the proximity of the samples in the spectral space for all of the 5 PCs. The results of this analysis are summarized in Table 1.

This analysis reflects the main source of variation in the dataset identified earlier: the contrast between the patients with different stages of caries development and patient without caries. We can see that the healthy patients are located separately from the group of the individuals with fissure and multiple caries. A separate group of the individuals with initial caries is observed, which indicates the prospect of searching for the biomarkers of the initial stages of caries.

The second most important source of variation in the dataset is the contrast between the oncology patients and those with different pathologies. An important source of variation in the data is the presence of a serious stage of periodontal disease. We note a rather pronounced gradation of the samples by their distance in space depending on the stage of this type of pathologies, which indicates the prospect of building predictive models for diagnosing this pathology in future research.

At the final stage, a cluster analysis on principal components was performed to assess the proximity of the molecular composition of the GCF samples in a multidimensional space. The method implemented in the FactoMineR package—Hierarchical Clustering on Principal Components (HCPC)—was used for this purpose. The number of clusters was selected based on the inertia loss criterion, as recommended by the package developers [80].

**Figure 11:**
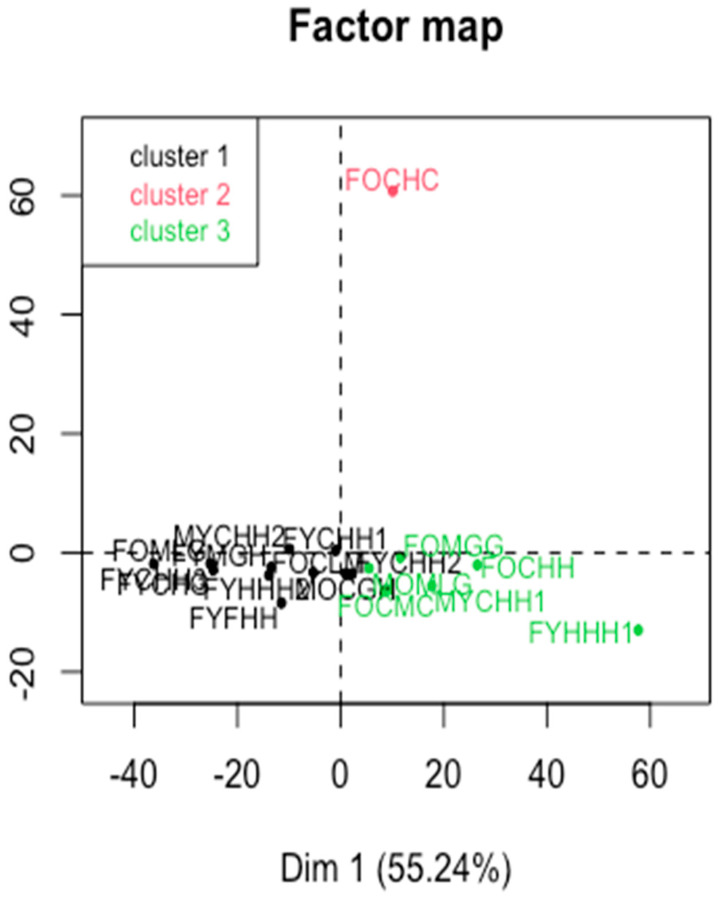
Hierarchical clustering on principal components.

As shown in Figure 11, the revealed clusters are not related to the factors, which may indicate that different characteristics of the molecular composition of the GCF samples are interrelated.

However, it is necessary to note the association of cluster 1 and 3 with the first component (v-test = −3.09, *p* < 0.001; v-test = 2.96, *p* < 0.001, respectively), the association of cluster 2 with the second component (v-test = 4.03, *p* = *p* < 0.0001). For the second cluster, the highest v-test values were observed for the features in the range of 1100–1150 cm^−1^, associated with the ν_s_ PO^2^- band, the carbohydrates, phosphates and the DNA derived structures [30,59,72] (test values are positive). The above facts obviously need further investigation.

We tested the clustering results using the latest Projection-Based Clustering package [81], which implements newly developed clustering methods that allowed us to estimate the presence of the cluster structure and apply non-linear dimensionality reduction methods prior to the clustering. We tested various methods and combinations of dimensionality reduction and clustering methods implemented in the package, and in the most cases all the samples were combined into one cluster, except for the FOCHC sample which formed a separate one. The reasons for this difference between the samples need further investigation.

The studies of the changes in the GCF molecular composition using FTIR revealed some differences in the biochemical profiles of the patients with different levels of caries development stage and periodontal status, as well as in the presence of comorbidities.

At the same time, the main changes observed in the GCF composition analysis are related to the functional groups correlated with the symmetric and the asymmetric stretches of the C-H groups CH_3_, CH_2_ attributable to the lipids and the fatty acids chains, >C=O carbonyl ester group (ester carbonyl), the changes in the structure of the amide bands Amid I, Amid II, Amid III, symmetrical and asymmetrical vibrations of PO^2^- referred to the phosphodiesters, nucleic acids, antibodies to nuclear antigens, and molecular bonds referred to the phosphates, as well as carbohydrates and the DNA derivatives of the structures in the low-frequency range.

It should be noted that the FTIR spectra of GCF are rather complex due to the large number of overlapping bands of the major components and compounds which appear in GCF.

## 3. Methods and Objects of Research

### 3.1. Experimental Design

Seventeen participants were selected for the study, from whom the gingival fluid samples were subsequently collected. The ethical approval for the study was obtained from the Ethics Committee of the N.N. Burdenko Voronezh State Medical University, and an informed consent was obtained from all the participants.

All of the study participants were of Caucasian race, men and women aged 25–60 years. At the time of the experiment, they were not taking any antibiotics, medications, did not smoke or drink alcohol. The clinical examination of the oral cavity for the study of the participants was performed in accordance with all the World Health Organization (WHO) rules and the recommendations provided by the Dental Clinic of the N.N. Burdenko Voronezh State Medical University. During the examination, the presence or absence of dental caries, periodontal diseases, and clinical picture (demographics and medical history) confirmed by entries in the personal medical record was registered for each patient. The classification of carious lesions was based on The International Caries Detection and Assessment System (ICDAS), and the patients were divided into the following groups: healthy, initial (ICDAS 1–2), fissure (ICDAS 2), multiple caries (ICDAS 3–4). The classification of periodontal status and disease severity was performed using the system recommended by the Center for Disease Control and Prevention/American Academy of Periodontology CDC-AAP Periodontitis classification. The patients were divided into the following groups: Healthy, Mild (gingivitis), Moderate (periodontitis, light and medium).

The respondents for the study were selected in such a way as to account for different combinations of the factors describing them (different demographics, caries development stages, periodontal status, presence/absence of concomitant chronic diseases).

### 3.2. Dataset Description

Table 2 presents a set of information about the study participants: demographics, caries development stage, periodontal status and medical history.

### 3.3. Collection of Gingival Fluid

Gingival fluid samples were collected at the Dental Clinic of the N.N. Burdenko Voronezh State Medical University between 10 a.m. and 12 a.m. to minimize the circadian rhythm. No stimulation was used during the gingival fluid collection. The collection was performed using microcapillaries that we had developed and successfully tested earlier [37,84].

Taking into account the experience described in a number of works where the capillary effect was used for sampling of macro-amounts of bioanalyte [85], in this study special tips were prepared for the GCF sampling. The microcapillary, 500 microns in diameter, was filled with homogenized fine potassium bromide (KBr) powder, which was sealed with a non-woven filter. Potassium bromide is an inert adsorbing agent for the investigated fluid and its choice as a filler is based on the absence of any absorption bands in a wide spectral range of IR-spectrum. During the gingival fluid collection, the microcapillary was connected to a sterilized syringe, which in turn was connected to a vacuum pump that allowed the collected fluid to flow into the microcapillary.

Prior to the collection of the GCF samples, the participants cleaned the oral cavity with a toothbrush and rinsed with clean water. Thirty minutes following the cleaning procedure and drying of the oral cavity with dry air from an oil-free compressor, the GCF was collected using a microcapillary. The timing was chosen based on the fact that GCF is released into the sulcus at a rate of ~3 µL/h in the absence of pathology, and in the presence of inflammation this value represents the concentration of metabolites in the serum which increases in volume as the inflammation progresses to ~44 µL/h [20]. To isolate the area of gingival fluid intake, the patient’s teeth on the vestibular and oral sides were covered with sterile cotton rolls.

Following the collection, the microcapillaries were immediately placed in sterile boxes and stored at −25 °C at a thermo container “SafePack”. The samples were transported to the Korean synchrotron and analyzed within 7 days after the collection. 

### 3.4. FTIR-Spectroscopy

FTIR spectra were obtained using the Infrared Synchrotron Radiation Beamline at PAL (Pohang, Republic of Korea), which provides a higher brightness and flux in the mid-infrared region, which allowed us to perform the reliable measurements of the GCF samples collected in microscopic volumes. A setup based on a Bruker Vertex 80/v vacuum FTIR spectrometer was used. All the synchrotron FTIR spectra were recorded within a spectral range of 3800–700 cm^−1^ using 4-cm^−1^ spectral resolution. Each sample (microcapillary contents) was divided into 5 parts, and the spectra were obtained from each part and subsequently averaged. Blackman-Harris 3-Term apodization, Mertz phase correction, and zero-filling factor of 2 were set as default acquisition parameters using OPUS 8 software suite (Bruker Optik GmbH, Ettlingen, Germany).

A MIRacle™ Single Reflection ATR with a ZnSe prism was used to analyze a small volume of the gingival fluid samples.

### 3.5. Spectra Processing

Spectra processing was performed at the Department of Solid-State Physics and Nanostructures (Voronezh State University). The standard initial processing of the FTIR data of GCF and potassium bromide powder involved smoothing the curves with a second-order Savitsky-Golay polynomial function (25 points). Further, the spectrum of potassium bromide powder was subtracted from the IR spectra of the gingival fluid samples to eliminate its possible effect on the features of spectral curves. The baseline correction was carried out by means of the OriginPro 2018 software package using the asymmetric least squares method for the data in the range from 3800–700 cm^−1^ with a threshold value of 0.01 and an asymmetric factor of about 0.001.

In this paper, we analyzed only the FTIR bands in the 3400–2800 cm^−1^ and 1800–870 cm^−1^ regions.

### 3.6. Multivariate Analysis of Spectral Data

Following the preliminary processing, the spectral data array was subjected (Psycholinguistic Textual Modelling Lab, Voronezh State Pedagogical University) to an exploratory analysis using the principal component analysis (PCA) with the subsequent analysis of the relationship between the extracted components and the factors that may influence the variation in the spectral dataset.

The use of PCA allowed us to reduce the dimensionality of the data, creating new variables—“principal components” (PC), which are a combination of the original variables and are a type of synthetic variables [86,87]. Multivariate analysis methods are robust in the sense that they provide a moderate trade-off between the large volume of initial data and its final aggregation. In this regard, PCA [57,86,88,89] is probably the most widely used multivariate statistical technique.

The spectral intensities at the given wave numbers were used as the features (variables), and the frequency position of the vibrational modes in a multidimensional space was analyzed, taking into account the patient’s personalized information. The dataset was a matrix of the spectral data from 17 samples.

Each specimen in the dataset represented the FTIR spectrum of a GCF sample. Subsequent analysis was performed in the bands of 3400–2800 cm^−1^ and 1800–870 cm^−1^.

A multivariate statistical analysis of the spectral data was implemented using the FactoMineR [56] and factoextra [90] R packages. They allowed us to analyze the relationship between the PCs and the metadata (the participants’ characteristics). The analysis and the visualization of the relationship between the characteristics of the participants were carried out using the R packages ggcorrplot and lsr. The tidyverse R package was also used to preprocess the data. 

The participants’ characteristics were represented as categorical variables with different number of the factor levels. Gender and age were considered as binary factors, the other respondents’ characteristics (related to their caries and periodontal status and anamnesis) were treated as multicategorical factor variables.

To investigate whether there was a relationship between the categorical variable of interest and the other categorical ones, we used the Chi-square test. The more significant it is (i.e., the smaller the *p*-values), the more doubtful is the hypothesis of the independence of variables (when conducting a chi-squared test of independence, the null hypothesis is independence, not dependence) [80].

To investigate the level of association between the factors, we used the Cramer’s V test calculated using the lsr package.

Further, a correlation analysis was conducted to examine the relationship between the levels of the factors. In this way, the relationships between the respondents’ different characteristics were investigated.

FactoMineR helps one to interprete the results of PCA. The catdes function of the FactoMineR package was used to analyze the relationship between the selected components and factors (see [91] for a detailed description of the function). The dimdes function of the FactoMineR was used to describe the selected components. For a supplementary categorical variable (i.e., one not participating in the analysis but used for the description), it performs a one-way analysis of variance with the coordinates of the individuals on the axis accounted for by means of the categorical variable. Then, for each category of the categorical variable, a student *t*-test is used to compare the average of the category with the general one (using the constraint Pi αi = 0, we test αi = 0). Then the *p*-value associated with this test is transformed into a normal quantile in order to take into account the information that the mean of the category is less or greater than 0 (we use the sign of the difference between the mean of the category and the overall one). This transformation named a v-test was introduced by Lebart, Morineau, and Piron [92].

For a quantitative variable, FactoMineR calculates the correlation coefficient between the variable and the coordinates of the individuals on the axis (Fs(i)). The correlation coefficients are calculated for all the variables, dimension by dimension. Then, FactoMineR performs tests for assessing the significance of each correlation coefficient and sorts the variables from the most correlated to the less correlated ones. Each dimension is then described by the variables. This is particularly useful for interpreting the dimensions when there are a lot of variables.

To identify the features that contribute to the components the most, the loadings were also calculated. Thus, we have established the associations between the variables (i.e., the intensities of the spectra at certain wave numbers) and the patients’ characteristics. In the analysis, the variables were not subjected to standardization, as they had already been processed before (see the section on the spectra processing). 

In the final step, a cluster analysis on principal components was performed using the FactomineR package, which produces a more robust cluster solution than that on raw data [80].

## 4. Limitations

A significant limitation of our study is a small sample size and the pooling of the infrequently repeated factors in a single respondent. However, one should note that our study is an exploratory one and it is directed at revealing the latent interrelations between the patients’ different characteristics which can be a source of biases in further studies and should thus be taken into account while determining the subgroups in a follow-up subgroup analysis.

## 5. Conclusions

In our exploratory study we sought to search for the associations between the two most common diseases of the oral cavity—dental caries and periodontal pathologies, taking into account an additional factors which describe personalized clinical picture (individual risk factors of the patient) based on the methods of multivariate data analysis of the molecular changes in the composition of human gingival crevicular fluid.

We examined the latent interrelations between different factors related to the patients that might have an effect on the spectral characteristics of the gingival crevicular fluid samples and therefore unless considered might result in various biases. 

For this purpose, a set of synchrotron infrared spectra of the GCF samples from the patients with different demographics, stages of dental caries development, periodontal pathologies and the presence/absence of concomitant chronic diseases was obtained and analyzed.

For the first time, using advanced multivariate data analysis techniques, an association between the stages of dental caries development and periodontal status has been studied, taking into account the patient’s personalized clinical picture.

By identifying the features (the vibrational modes in the FTIR spectra) that contribute to the differentiation of the spectral dataset the most, and by taking into account the interrelationships of the patients’ characteristics, we have been able to match the specific biological markers (specific molecular groups) to the two factors of interest—two types of oral pathologies—for the first time.

The revealed changes in the quantitative and qualitative mode composition in the FTIR spectra of the GCF samples from the patients with different dental caries development stages and in the presence of periodontal diseases confirm the difficulty of identifying patient-specific spectral information. At the same time, different periodontal pathologies are more closely associated with the patients’ other characteristics than the stages of caries development.

The performed multivariate analysis of the spectral dataset indicates the need to take into account not only the co-occurrence of oral diseases, but also some other factors. If not considered (which is typical of lots of studies in this area) they may lead to misinterpretation and consequently the loss of data when searching for the biological markers of certain oral diseases. 

A code in R to reproduce our analysis is available by request. The methodology proposed in the paper can be applied by other researchers in their preliminary examinations for performing the predefined subgroup analysis before conducting large-scale clinical examinations.

The future direction of this study involves the construction of the classifiers based on the obtained data using the Partial Least Squares Discriminant Analysis (PLS-DA) algorithm which is specially designed to classify multivariate data even under conditions of a small sample size and is resistant to collinearity. The subject of the classification will be presence/absence of caries and periodontal status. An error analysis of the classifier output will be performed taking into account additional information about the study participants. This algorithm will be implemented in two variants—without and with pre-selection of the features.

## Figures and Tables

**Table 1:** Distance of the samples in a 5-dimensional space.

Factor	Factor Level	Dist
Gender	Male	2.09
	Female	7.33
Age	Young	5.52
	Old	6.90
Dental caries	Healthy	29.19
	Initial	17.43
	Fissure	7.26
	Multiple	3.90
Periodontum	Healthy	3.728241
	Gingivitis	7.590230
	Light	11.756492
	Moderate	20.138446
Anamnesis	Healthy	5.064526
	Gastrointestinal	9.235628
	Multiple	14.845186
	Cancer	30.593537

**Table 2:** Participants’ information. The participants were divided into two groups with respect to their age: young (25–44 y.o.; Y) and old (45–60 y. o.; O) as recommended by the WHO [82,83]. The caries status was classified into healthy (H), initial (C), multiple (M), and fissure caries (F). Further, the participants were divided into groups with a healthy periodontal condition (H), with gingivitis (G), light and moderate periodontitis (L and M, accordingly). Finally, the clinical picture column (anamnesis) indicates the absence (H) or the presence of comorbidities such as gastrointestinal illnesses (G), cancer (C) or multiple diseases (M).

Sample	Name of Participants	Gender	Age	Caries Status	Periodontal Status	Anamnesis
1	FYHHH	F	Y	H	H	H
2	FYCHH	F	Y	C	H	H
3	FYCHH	F	Y	C	H	H
4	FOCHH	F	O	C	H	H
5	MYCGG	M	Y	C	G	G
6	MOCLH	M	O	C	L	H
7	FOCMC	F	O	C	M	C
8	FYCHG	F	Y	C	H	G
9	MYCLH	M	Y	C	L	H
10	FYCHM	F	Y	C	H	M
11	FOMLG	F	O	M	L	G
12	MOMLG	M	O	M	L	G
13	FOMHC	F	O	M	H	C
14	FOMLM	F	O	M	L	M
15	FOMLG	F	O	M	L	G
16	FYMLM	F	Y	M	L	M
17	FYFHH	F	Y	F	H	H

## Data Availability

The data that support the findings of this study are available from the corresponding author upon reasonable request.

## References

[1] Barranca-Enríquez A., Romo-González T. (2022). Your Health Is in Your Mouth: A Comprehensive View to Promote General Wellness. Front. Oral Health.

[2] World Health Organization (2023). Region Global Oral Health Status Report: Towards Universal Health Coverage for Oral Health by 2030: Summary of the WHO European Region. Global Oral Health Status Report: Towards Universal Health Coverage for Oral Health by 2030: Summary of the WHO European Region.

[3] Baima G., Shin H.-S., Arrica M., Laforí A., Cordaro M., Romandini M. (2023). The Co-Occurrence of the Two Main Oral Diseases: Periodontitis and Dental Caries. Clin. Oral Investig..

[4] Yu L.X., Wang X., Feng X.P., Tai B.J., Hu D.Y., Wang B., Wang C.X., Zheng S.G., Liu X.N., Rong W.S. (2021). The Relationship between Different Types of Caries and Periodontal Disease Severity in Middle-Aged and Elderly People: Findings from the 4th National Oral Health Survey of China. BMC Oral Health.

[5] Fraihat N., Madae’en S., Bencze Z., Herczeg A., Varga O. (2019). Clinical Effectiveness and Cost-Effectiveness of Oral-Health Promotion in Dental Caries Prevention among Children: Systematic Review and Meta-Analysis. Int. J. Environ. Res. Public. Health.

[6] Yoshihara K., Nagaoka N., Nakamura A., Hara T., Hayakawa S., Yoshida Y., Van Meerbeek B. (2020). Three-Dimensional Observation and Analysis of Remineralization in Dentinal Caries Lesions. Sci. Rep..

[7] Boroumand M., Olianas A., Cabras T., Manconi B., Fanni D., Faa G., Desiderio C., Messana I., Castagnola M. (2021). Saliva, a Bodily Fluid with Recognized and Potential Diagnostic Applications. J. Sep. Sci..

[8] García-Godoy F., Hicks M.J. (2008). Maintaining the Integrity of the Enamel Surface: The Role of Dental Biofilm, Saliva and Preventive Agents in Enamel Demineralization and Remineralization. J. Am. Dent. Assoc..

[9] Hicks J., Garcia-Godoy F., Flaitz C. (2004). Biological Factors in Dental Caries: Role of Saliva and Dental Plaque in the Dynamic Process of Demineralization and Remineralization (Part 1). J. Clin. Pediatr. Dent..

[10] Aruni A.W., Dou Y., Mishra A., Fletcher H.M. (2015). The Biofilm Community: Rebels with a Cause. Curr. Oral Health Rep..

[11] Durand R., Roufegarinejad A., Chandad F., Rompré P.H., Voyer R., Michalowicz B.S., Emami E. (2019). Dental Caries Are Positively Associated with Periodontal Disease Severity. Clin. Oral Investig..

[12] Bowen W.H., Burne R.A., Wu H., Koo H. (2018). Oral Biofilms: Pathogens, Matrix, and Polymicrobial Interactions in Microenvironments. Trends Microbiol..

[13] Colombo A.P.V., Tanner A.C.R. (2019). The Role of Bacterial Biofilms in Dental Caries and Periodontal and Peri-Implant Diseases: A Historical Perspective. J. Dent. Res..

[14] Bertolini M., Costa R., Barão V., Villar C.C., Retamal-Valdes B., Feres M., Silva Souza J. (2022). Oral Microorganisms and Biofilms: New Insights to Defeat the Main Etiologic Factor of Oral Diseases. Microorganisms.

[15] Portaccio M., d’Apuzzo F., Perillo L., Grassia V., Errico S., Lepore M. (2019). Infrared Microspectroscopy Characterization of Gingival Crevicular Fluid during Orthodontic Treatment. J. Mol. Struct..

[16] Seredin P., Goloshchapov D., Ippolitov Y., Vongsvivut P. (2018). Pathology-Specific Molecular Profiles of Saliva in Patients with Multiple Dental Caries—Potential Application for Predictive, Preventive and Personalised Medical Services. EPMA J..

[17] Lips A., Antunes L.S., Antunes L.A., Pintor A.V.B., dos Santos D.A.B., Bachinski R., Küchler E.C., Alves G.G. (2017). Salivary Protein Polymorphisms and Risk of Dental Caries: A Systematic Review. Braz. Oral Res..

[18] Kripal K., Bhavanam S., Anuroopa P., Kumar P., Chandrasekaran K., Paul P. (2018). Comparision of the Microbial Count in Supragingival Plaque, Gingival Crevicular Blood and Saliva Samples Immediately after Diode Laser (970 ± 15 Nm) Application in Chronic Periodontitis Patients: A Randomized Controlled Split Mouth Clinical Trial. Dentistry.

[19] Delrue C., De Bruyne S., Speeckaert M.M. (2023). Unlocking the Diagnostic Potential of Saliva: A Comprehensive Review of Infrared Spectroscopy and Its Applications in Salivary Analysis. J. Pers. Med..

[20] Saloom H.F., Carpenter G.H., Bergmeier L.A. (2018). Saliva and Gingival Crevicular Fluid: Contributions to Mucosal Defense. Oral Mucosa in Health and Disease: A Concise Handbook.

[21] Barros S.P., Williams R., Offenbacher S., Morelli T. (2016). Gingival Crevicular Fluid as a Source of Biomarkers for Periodontitis. Periodontology 2000.

[22] Bibi T., Khurshid Z., Rehman A., Imran E., Srivastava K.C., Shrivastava D. (2021). Gingival Crevicular Fluid (GCF): A Diagnostic Tool for the Detection of Periodontal Health and Diseases. Molecules.

[23] Syed S., Kankara V., Pathakota K., Krishnan P., Mishra A. (2020). Evaluation of Deoxypyridinoline Levels in Gingival Crevicular Fluid and Serum as Alveolar Bone Loss Biomarker in Patients with Periodontitis. J. Indian Soc. Periodontol..

[24] d’Apuzzo F., Nucci L., Delfino I., Portaccio M., Minervini G., Isola G., Serino I., Camerlingo C., Lepore M. (2021). Application of Vibrational Spectroscopies in the Qualitative Analysis of Gingival Crevicular Fluid and Periodontal Ligament during Orthodontic Tooth Movement. J. Clin. Med..

[25] Nazar Majeed Z., Philip K., Alabsi A.M., Pushparajan S., Swaminathan D. (2016). Identification of Gingival Crevicular Fluid Sampling, Analytical Methods, and Oral Biomarkers for the Diagnosis and Monitoring of Periodontal Diseases: A Systematic Review. Dis. Markers.

[26] Toczewska J., Baczyńska D., Zalewska A., Maciejczyk M., Konopka T. (2023). The mRNA Expression of Genes Encoding Selected Antioxidant Enzymes and Thioredoxin, and the Concentrations of Their Protein Products in Gingival Crevicular Fluid and Saliva during Periodontitis. Dent. Med. Probl..

[27] Yu J.C., Khodadadi H., Baban B. (2019). Innate Immunity and Oral Microbiome: A Personalized, Predictive, and Preventive Approach to the Management of Oral Diseases. EPMA J..

[28] Ranc V., Žižka R., Chaloupková Z., Ševčík J., Zbořil R. (2018). Imaging of Growth Factors on a Human Tooth Root Canal by Surface-Enhanced Raman Spectroscopy. Anal. Bioanal. Chem..

[29] Júnior C., Cesar P., Strixino J.F., Raniero L., Júnior C., Cesar P., Strixino J.F., Raniero L. (2015). Analysis of Saliva by Fourier Transform Infrared Spectroscopy for Diagnosis of Physiological Stress in Athletes. Res. Biomed. Eng..

[30] Lopes J., Correia M., Martins I., Henriques A.G., Delgadillo I., da Cruz e Silva O., Nunes A. (2016). FTIR and Raman Spectroscopy Applied to Dementia Diagnosis Through Analysis of Biological Fluids. J. Alzheimers Dis..

[31] Bottoni U., Tiriolo R., Pullano S.A., Dastoli S., Amoruso G.F., Nisticò S.P., Fiorillo A.S. (2016). Infrared Saliva Analysis of Psoriatic and Diabetic Patients: Similarities in Protein Components. IEEE Trans. Biomed. Eng..

[32] Gupta G. (2013). Gingival Crevicular Fluid as a Periodontal Diagnostic Indicator- II: Inflammatory Mediators, Host-Response Modifiers and Chair Side Diagnostic Aids. J. Med. Life.

[33] Mah J., Prasad N. (2004). Dentine Phosphoproteins in Gingival Crevicular Fluid during Root Resorption. Eur. J. Orthod..

[34] Guo L., Shi W. (2013). Salivary Biomarkers for Caries Risk Assessment. J. Calif. Dent. Assoc..

[35] Dias C.d.A. (2013). Salivary Biomarkers of Dental Caries.

[36] Camerlingo C., Portaccio M., d’Apuzzo F., Nucci L., Perillo L., Lepore M. (2022). μ-FTIR, μ-Raman, and SERS Analysis of Amide I Spectral Region in Oral Biofluid Samples during Orthodontic Treatment. Sensors.

[37] Seredin P., Goloshchapov D., Ippolitov Y., Vongsvivut J. (2020). Comparative Analysis of Dentine and Gingival Fluid Molecular Composition and Protein Conformations during Development of Dentine Caries: A Pilot Study. Vib. Spectrosc..

[38] Abdelkawi S., El Saeid A., El-Rashedi A., Abd-Eldaiem M. (2018). Photobiomodulation Therapy for Diabetic Macular Edema: Fourier Transform Infrared Spectroscopy Study. J. Arab Soc. Med. Res..

[39] Fujii S., Sato S., Fukuda K., Okinaga T., Ariyoshi W., Usui M., Nakashima K., Nishihara T., Takenaka S. (2016). Diagnosis of Periodontal Disease from Saliva Samples Using Fourier Transform Infrared Microscopy Coupled with Partial Least Squares Discriminant Analysis. Anal. Sci. Int. J. Jpn. Soc. Anal. Chem..

[40] Bellisola G., Sorio C. (2012). Infrared Spectroscopy and Microscopy in Cancer Research and Diagnosis. Am. J. Cancer Res..

[41] Titus J., Ghimire H., Viennois E., Merlin D., Perera A.G.U. (2018). Protein Secondary Structure Analysis of Dried Blood Serum Using Infrared Spectroscopy to Identify Markers for Colitis Screening. J. Biophotonics.

[42] Babot-Marquillas C., Sánchez-Martín M.-J., Amigo J.M., Yousef I.H., Valido I., Boada R., Valiente M. (2022). Tooth Whitening, Oxidation or Reduction? Study of Physicochemical Alterations in Bovine Enamel Using Synchrotron Based Micro-FTIR. Dent. Mater..

[43] Seredin P., Goloshchapov D., Kashkarov V., Khydyakov Y., Nesterov D., Ippolitov I., Ippolitov Y., Vongsvivut J. (2022). Development of a Hybrid Biomimetic Enamel-Biocomposite Interface and a Study of Its Molecular Features Using Synchrotron Submicron ATR-FTIR Microspectroscopy and Multivariate Analysis Techniques. Int. J. Mol. Sci..

[44] Seredin P., Goloshchapov D., Kashkarov V., Ippolitov Y., Ippolitov I., Vongsvivut J. (2021). To the Question on the Use of Multivariate Analysis and 2D Visualisation of Synchrotron ATR-FTIR Chemical Imaging Spectral Data in the Diagnostics of Biomimetic Sound Dentin/Dental Composite Interface. Diagnostics.

[45] Seredin P., Goloshchapov D., Ippolitov Y., Vongsvivut J. (2020). Development of a New Approach to Diagnosis of the Early Fluorosis Forms by Means of FTIR and Raman Microspectroscopy. Sci. Rep..

[46] Seredin P., Kashkarov V., Lukin A., Ippolitov Y., Julian R., Doyle S. (2013). Local Study of Fissure Caries by Fourier Transform Infrared Microscopy and X-Ray Diffraction Using Synchrotron Radiation. J. Synchrotron Radiat..

[47] Belstrøm D., Jersie-Christensen R.R., Lyon D., Damgaard C., Jensen L.J., Holmstrup P., Olsen J.V. (2016). Metaproteomics of Saliva Identifies Human Protein Markers Specific for Individuals with Periodontitis and Dental Caries Compared to Orally Healthy Controls. PeerJ.

[48] Bel’skaya L.V., Sarf E.A., Solomatin D.V. (2020). Age and Gender Characteristics of the Infrared Spectra of Normal Human Saliva. Appl. Spectrosc..

[49] Zhang Y., Ren L., Wang Q., Wen Z., Liu C., Ding Y. (2022). Raman Spectroscopy: A Potential Diagnostic Tool for Oral Diseases. Front. Cell. Infect. Microbiol..

[50] Bel’skaya L.V., Sarf E.A. (2021). Biochemical Composition and Characteristics of Salivary FTIR Spectra: Correlation Analysis. J. Mol. Liq..

[51] Caixeta D.C., Carneiro M.G., Rodrigues R., Alves D.C.T., Goulart L.R., Cunha T.M., Espindola F.S., Vitorino R., Sabino-Silva R. (2023). Salivary ATR-FTIR Spectroscopy Coupled with Support Vector Machine Classification for Screening of Type 2 Diabetes Mellitus. Diagnostics.

[52] Durlik-Popińska K., Żarnowiec P., Konieczna-Kwinkowska I., Lechowicz Ł., Gawęda J., Kaca W. (2021). Correlations between Autoantibodies and the ATR-FTIR Spectra of Sera from Rheumatoid Arthritis Patients. Sci. Rep..

[53] Sanchez-Brito M., Vazquez-Zapien G.J., Luna-Rosas F.J., Mendoza-Gonzalez R., Martinez-Romo J.C., Mata-Miranda M.M. (2022). Attenuated Total Reflection FTIR Dataset for Identification of Type 2 Diabetes Using Saliva. Comput. Struct. Biotechnol. J..

[54] Giorgini E., Balercia P., Conti C., Ferraris P., Sabbatini S., Rubini C., Tosi G. (2013). Insights on Diagnosis of Oral Cavity Pathologies by Infrared Spectroscopy: A Review. J. Mol. Struct..

[55] Derruau S., Gobinet C., Mateu A., Untereiner V., Lorimier S., Piot O. (2019). Shedding Light on Confounding Factors Likely to Affect Salivary Infrared Biosignatures. Anal. Bioanal. Chem..

[56] Lê S., Josse J., Husson F. (2008). FactoMineR: An R Package for Multivariate Analysis. J. Stat. Softw..

[57] Baker M.J., Hussain S.R., Lovergne L., Untereiner V., Hughes C., Lukaszewski R.A., Thiéfin G., Sockalingum G.D. (2016). Developing and Understanding Biofluid Vibrational Spectroscopy: A Critical Review. Chem. Soc. Rev..

[58] Orphanou C.-M. (2015). The Detection and Discrimination of Human Body Fluids Using ATR FT-IR Spectroscopy. Forensic Sci. Int..

[59] Matthäus C., Bird B., Miljković M., Chernenko T., Romeo M., Diem M. (2008). Infrared and Raman Microscopy in Cell Biology. Methods Cell Biol..

[60] Seredin P., Goloshchapov D., Kashkarov V., Ippolitov Y., Bambery K. (2016). The Investigations of Changes in Mineral–Organic and Carbon–Phosphate Ratios in the Mixed Saliva by Synchrotron Infrared Spectroscopy. Results Phys..

[61] Seredin P., Goloshchapov D., Kashkarov V., Nesterov D., Ippolitov Y., Ippolitov I., Vongsvivut J. (2022). Effect of Exo/Endogenous Prophylaxis Dentifrice/Drug and Cariogenic Conditions of Patient on Molecular Property of Dental Biofilm: Synchrotron FTIR Spectroscopic Study. Pharmaceutics.

[62] Naumann D., Roberts G.C.K. (2013). Infrared Spectroscopy of Cells, Tissues, and Biofluids. Encyclopedia of Biophysics.

[63] Scherdin-Almhöjd U. (2017). Identification of Esters in Carious Dentine Staining and Chemo-Mechanical Excavation. Ph.D. Thesis.

[64] Almhöjd U.S., Norén J.G., Arvidsson A., Nilsson Å., Lingström P. (2014). Analysis of Carious Dentine Using FTIR and ToF-SIMS. Oral Health Dent. Manag..

[65] Larmas M. (1972). A Chromatographic and Histochemical Study of Nonspecific Esterases in Human Carious Dentine. Arch. Oral Biol..

[66] Yang H., Yang S., Kong J., Dong A., Yu S. (2015). Obtaining Information about Protein Secondary Structures in Aqueous Solution Using Fourier Transform IR Spectroscopy. Nat. Protoc..

[67] Huang Y.-T., Liao H.-F., Wang S.-L., Lin S.-Y. (2016). Glycation and Secondary Conformational Changes of Human Serum Albumin: Study of the FTIR Spectroscopic Curve-Fitting Technique. AIMS Biophys..

[68] Stuart B. (2004). Infrared Spectroscopy: Fundamentals and Applications.

[69] Depciuch J., Sowa-Kućma M., Nowak G., Dudek D., Siwek M., Styczeń K., Parlińska-Wojtan M. (2016). Phospholipid-Protein Balance in Affective Disorders: Analysis of Human Blood Serum Using Raman and FTIR Spectroscopy. A Pilot Study. J. Pharm. Biomed. Anal..

[70] Kong J., Yu S. (2007). Fourier Transform Infrared Spectroscopic Analysis of Protein Secondary Structures. Acta Biochim. Biophys. Sin..

[71] Seredin P.V., Goloshchapov D.L., Ippolitov Y.A., Kalivradzhiyan E.S. (2018). Does Dentifrice Provide the Necessary Saturation of Ions in Oral Fluids to Favour Remineralisation?. Russ. Open Med. J..

[72] Makhnii T., Ilchenko O., Reynt A., Pilgun Y., Kutsyk A., Krasnenkov D., Ivasyuk M., Kukharskyy V. (2016). Age-Related Changes in FTIR and Raman Spectra of Human Blood. Ukr. J. Phys..

[73] Tajmir-Riahi H.A., N’soukpoe-kossi C.N., Joly D. (2009). Structural Analysis of Protein-DNA and Protein-RNA Interactions by FTIR Spectroscopy. Adv. Biomed. Spectrosc..

[74] Elkins K.M. (2011). Rapid Presumptive “Fingerprinting” of Body Fluids and Materials by ATR FT-IR Spectroscopy. J. Forensic Sci..

[75] Xiang X.M., Liu K.Z., Man A., Ghiabi E., Cholakis A., Scott D.A. (2010). Periodontitis-Specific Molecular Signatures in Gingival Crevicular Fluid. J. Periodontal Res..

[76] Seredin P., Goloshchapov D., Kashkarov V., Lukin A., Peshkov Y., Ippolitov I., Ippolitov Y., Litvinova T., Vongsvivut J., Chae B. (2023). Changes in Dental Biofilm Proteins’ Secondary Structure in Groups of People with Different Cariogenic Situations in the Oral Cavity and Using Medications by Means of Synchrotron FTIR-Microspectroscopy. Int. J. Mol. Sci..

[77] Kim S., Song Y., Kim S., Kim S., Na H., Lee S., Chung J., Kim S. (2023). Identification of a Biomarker Panel for Diagnosis of Early Childhood Caries Using Salivary Metabolic Profile. Metabolites.

[78] Fidalgo T.K.S., Freitas-Fernandes L.B., Angeli R., Muniz A.M.S., Gonsalves E., Santos R., Nadal J., Almeida F.C.L., Valente A.P., Souza I.P.R. (2013). Salivary Metabolite Signatures of Children with and without Dental Caries Lesions. Metabolomics.

[79] Cattell R.B. (1966). The Scree Test for The Number of Factors. Multivar. Behav. Res..

[80] Husson F., Lê S., Pagès J. (2017). Exploratory Multivariate Analysis by Example Using R.

[81] Thrun M.C. (2018). Projection-Based Clustering through Self-Organization and Swarm Intelligence.

[82] Department of Economic and Social Affairs (2020). World Population Ageing 2019.

[83] Ahmad O.B., Boschi Pinto C., Lopez A.D. (2001). Age Standardization of Rates: A New WHO Standard. GPE Discuss. Pap. Ser. No 31.

[84] Seredin P., Goloshchapov D., Ippolitov Y. (2019). Jitraporn Vongsvivut Spectroscopic Signature of the Pathological Processes of Carious Dentine Based on FTIR Investigations of the Oral Biological Fluids. Biomed. Opt. Express.

[85] Reddy N., Deepa A., Madhu Babu D., Chandra N., Subba Reddy C., Kumar A. (2014). Estimation of Tissue Inhibitor of Matrix Metalloproteinase-1 Levels in Gingival Crevicular Fluid in Periodontal Health, Disease and after Treatment. J. Indian Soc. Periodontol..

[86] Abdi H., Williams L.J. (2010). Principal Component Analysis. WIREs Comput. Stat..

[87] Martin F.L., Dickinson A.W., Saba T., Bongers T., Singh M.N., Bury D. (2023). ATR-FTIR Spectroscopy with Chemometrics for Analysis of Saliva Samples Obtained in a Lung-Cancer-Screening Programme: Application of Swabs as a Paradigm for High Throughput in a Clinical Setting. J. Pers. Med..

[88] González-Solís J.L., Martínez-Cano E., Magaña-López Y. (2015). Early Detection of Dental Fluorosis Using Raman Spectroscopy and Principal Component Analysis. Lasers Med. Sci..

[89] Rohman A., Windarsih A., Lukitaningsih E., Rafi M., Betania K., Fadzillah N.A. (2020). The Use of FTIR and Raman Spectroscopy in Combination with Chemometrics for Analysis of Biomolecules in Biomedical Fluids: A Review. Biomed. Spectrosc. Imaging.

[90] Kassambara A., Mundt F. Factoextra: Extract and Visualize the Results of Multivariate Data Analyses 2020. http://www.sthda.com/english/rpkgs/factoextra.

[91] FactoMineR: Catdes. http://factominer.free.fr/factomethods/categories-description.html.

[92] Lebart L., Morineau A., Piron M. (2000). Statistique Exploratoire Multidimensionnelle.

